# Retrospective Analysis of Microbial Colonization Patterns in Central Venous Catheters, 2013–2017

**DOI:** 10.1155/2019/8632701

**Published:** 2019-09-17

**Authors:** Yu He, Huihan Zhao, Yan Wei, Xiao Gan, Ying Ling, Yanping Ying

**Affiliations:** ^1^Department of Clinical Laboratory, The First Affiliated Hospital of Guangxi Medical University, No. 6., Shuangyong Road, 530021 Nanning, Guangxi, China; ^2^Department of Nursing, The First Affiliated Hospital of Guangxi Medical University, No. 6., Shuangyong Road, 530021 Nanning, Guangxi, China; ^3^Department of Medical Oncology, The First Affiliated Hospital of Guangxi Medical University, No. 6., Shuangyong Road, 530021 Nanning, Guangxi, China

## Abstract

**Objectives:**

This study was performed to provide epidemiological information on microbial colonization in central venous catheters (CVCs).

**Methods:**

CVCs submitted to Medical Microbiology Laboratory from January 1, 2013, through October 1, 2017, which met our criteria would be included for analysis. Quantitative culture was used for CVCs. The results of culture and related information on CVCs were collected and recorded in detail. The prevalence was calculated, and related factors were analyzed statistically.

**Results:**

A total of 2020 CVCs were submitted for culture and eligible for analysis. Positive microbial culture occurred in 379 catheters with 18.7% (379 of 2020) prevalence of colonization. There were 23 microbial genera and 45 organisms detected. Among the isolated organisms, there were 39 kinds of isolated bacteria and 6 kinds of isolated fungi. *Acinetobacter* (19.8%) predominated in total isolated microorganisms, followed by *Staphylococcus epidermidis* (11.3%) and *Candida albicans* (10.3%). There were no significant differences in isolated organisms and fungal species between different sexes (*X*^2^ = 2.365, *P* = 0.50). Conversely, there were significant differences in isolated bacterial and fungal species between different wards and years (*X*^2^ = 124.046, *P* = 0.000; *X*^2^ = 77.064, *P* = 0.000). A total of 107 (5.3%, 107/2020) CVCs were associated with a diagnosis of central line-associated bloodstream infection (CLABSI). The most common organisms in causing CLABSI were *Acinetobacter* (23.4%), *S. aureus* (13.1%), and *Candida albicans* (12.1%).

**Conclusion:**

The prevalence of microbial colonization in CVCs is still significant and even has gradually changed over time. The study provides a new view of microbial colonization pattern in CVCs and a prevalence of CLABSI, which will facilitate catheter-related infection prevention and control in clinic.

## 1. Introduction

Intravascular catheters (IVCs) as medical devices are ubiquitous in health care setting. Most hospitalized patients benefit from intravascular catheters used to monitor haemodynamic status and administer drug, fluids, and parenteral nutrition. More than two billion intravascular devices are inserted globally each year [1]. Central venous catheter (CVC) as a common type of IVC and crucial device has been widely used in critical patients and oncology patients. CVC indwell promotes effective treatment and avoids the pain of repeated punctures for patients. Unfortunately, CVCs also carry unintended complications, such as infection and thrombosis, which are not rare. Infectious complications, especially central line-associated bloodstream infection (CLABSI), are potentially associated with poor patient outcomes, high morbidity and mortality, increasing hospitalization, and hospital costs [2].

The mean rate of CLABSIs in acute-care hospital units in the United States ranges from zero to 2.9/1000 CVC days depending on the type of unit [3]. Günther et al. reported that overall infectious complication's incidence rate was 14.5/1000 catheter days; in addition, catheter-tip colonization (14.2/1000 catheter days) was the most common [4]. CVC insertion allows an entrance for colonization of pathogenic bacteria. Pathogenic bacteria adhere successfully on the surface of the device in 24 hours, and host tissue cells and pathogenic bacteria compete to present on the surface of device. If the bacteria adhere successfully, biofilm formation would be initiated. The biofilm formation makes resistance to common antibiotics [5, 6] and evacuates adhered bacteria difficultly. So it becomes a major source of catheter-related blood infection and causes critical challenge for health care. In the United States, CLABSI accounts for an estimated 28,000 deaths and up to $2.3 billion annually [3, 7]. In China, the average economic loss per case of CLABSI is about ¥30713 [8], which makes it become the most costly form of health care-associated infections.

With a wide application of CVCs, the characteristics of microorganisms present diversity. 8 microbial phyla were reported, and 136 diverse microbial genera were detected on the IVC surfaces in children. *Staphylococcus* and *Streptococcus* were the most common [9]. However, a study form Spain showed *Gram-positive cocci* (68.4%) accounted for most episodes, followed by *yeasts* (26.3%) and *Gram-negative bacilli* (5.3%) in colonized catheters [10]. There were also studies, respectively, reporting that the predominant positive microorganisms were *coagulase-negative staphylococci* [11] and *S. aureus* [12]. Thus, the prevalence of microbial colonization in CVCs in different studies varies widely. Moreover, with the appearance of multidrug-resistant pathogens, the treatment of catheter-related infection and choice of antibiotics become more difficult. So characteristics and distribution of colonization of microorganisms on the catheters' surface need to be timely and accurately understood and updated as to guide clinical practice. Our study based on clinic presented distribution characteristics of microorganisms on the CVC surface, as to provide a reference for prevention and treatment of catheter-related infection in clinic.

## 2. Materials and Methods

A retrospective study over near 5 years (from January 2013 through October 2017) was carried out in a tertiary, general hospital in Guangxi, China. CVCs submitted to the clinical laboratory for culture during the period were objects of the study. Each catheter is an independent subject. The methods of catheter insertion did not standardize. All removed CVCs were not mandatory to submit for culture. Clinicians decided whether to culture the CVCs based on individual and clinical conditions. As we were only interested in the culture results of all submitted CVCs, all submitted CVC samples for culture in the laboratory were included. However, CVCs with incomplete culture information were excluded.

### 2.1. Catheter Tip Culture

Catheter removal and transport to the laboratory were standardized. All cultured catheters were removed using sterile gloves after the insertion site had been thoroughly cleaned with 2% povidone-iodine which are under aseptic conditions. The distal 2-3 cm of the catheter tip was cut with a sterile surgical scissors and put into a separate and labeled sterile container and then transported to the Medical Microbiology Laboratory in 2 hours for examination. All catheters tips planned to culture were cultured by the roll-plate culture method. The catheter tips were removed carefully using sterile forceps and then directly used to inoculate onto chocolate agar with 5% sheep blood agar in the laboratory. The catheter tip was rolled across the plate 2-3 times. The plates containing the catheter were incubated in 5% CO_2_ at 35–37°C for 18–24 hours, after which the number of organisms in the plates were evaluated quantitatively. A minimum of 15 colony-forming units (CFU) in each plate was considered as a positive catheter-tip culture, after which bacterial and fungal identification was performed using biochemical systems (VITEK 2 Compact, bioMerieux, France).

### 2.2. Blood Culture

During the 5 years, the blood culture results were reviewed and analyzed for each case from whom a CVC was collected and cultured as to analyze any relationship between the two types of culture. Blood culture was carried out by BacT-ALERT 3D120.

### 2.3. Data Collection

Some factors such as patient's sociodemographic characteristics, different wards, types of CVCs, positive blood culture results, and diagnosis of CLABSI were collected through the electronic medical record system.

### 2.4. Data Analysis

SPSS version 17.0 software package (Chicago, IL, USA) was used to input and analyze the data. Positive catheter tip, blood culture rates, and cultured fungal and bacterial epidemiological characteristics were evaluated. Fisher's exact test or chi-square test was used to test if differences existed between different related factors. *P* < 0.05 was considered significant.

## 3. Results

A total of 2020 CVCs were submitted for culture and eligible for analysis. There was no CVC excluded. The basic characteristics of patients with the CVCs are shown in Table 1.

Positive microbial culture occurred in 379 catheters with prevalence of microbial colonization of 18.7% (379 of 2020). There were 23 microbial genera and 45 organisms detected. Among the 45 isolated organisms, there were 39 isolated bacteria and 6 isolated fungi. Gram-negative bacteria with 44.4% predominated among the isolated bacteria. The most common Gram-negative bacteria were *Acinetobacter* (19.8%) followed by *Pseudomonas* (9.8%). Of Gram-positive bacteria (40.1%), *S. epidermidis* (11.3%) and *S. haemolyticus* (9.2%) were the most common. In fungi, *Candida albicans* with 10.3% was predominate. A total of 107 (5.3%, 107/2020) CVCs were associated with a diagnosis of CLABSI. The most common organisms in causing CLABSI were *Acinetobacter* (23.4%), *S. aureus* (13.1%), and *Candida albicans* (12.1%). The compositions of isolated organisms from CVCs and blood cultures are listed in Table 2.

**Figure 1:**
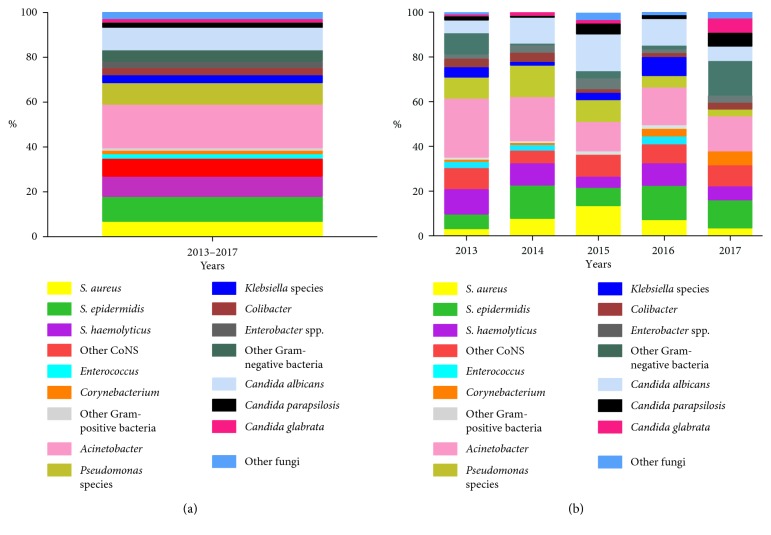
Bacterial and fungal species isolated from 2020 CVCs in different years. CVC, central venous catheter; CoNS, coagulase-negative staphylococci (a) The total composition of isolated organisms on 2020 CVCs surfaces; (b) the composition of isolated organisms on CVC surface in different years. The changes of proportion of color block in (b) indicate the proportion of Gram-negative bacteria decreased gradually from 2013 to 2017 and fungus with diversified internal composition was presented accompanied by the proportion of non-*Candida albicans* increasing in 2017.

There were no significant differences in isolated organisms and fungal species that were compared between different sexes (*X*^2^ = 2.365, *P*=0.50). Conversely, there were significant differences in isolated organisms and fungal species between different wards and years (*X*^2^ = 124.046, *P*=0.000; *X*^2^ = 77.064, *P*=0.000). The information on bacterial and fungal species isolated in different years and wards is listed in Table 3 and Figure 1.

## 4. Discussion

In the study, positive microbiologic culture in 18.4% and 45 types of microorganisms (39 bacterial and 6 fungal species) isolated is shown. From this study, *Acinetobacter* predominated in total isolated microorganisms, followed by *S. epidermidis* and *Candida albicans*. Comparing the prevalence of microbial colonization of 18.4%, several studies using molecular analysis has showed a higher colonization rate, and even almost all implanted venous access devices were colonized in recent years [12–14], which may be attributed to different analysis techniques. Molecular analysis with a higher detection rate should be developed and applied in clinic. In addition, it is similar with a study including 4281 CVCs in China which reported *Acinetobacter baumannii* was the most common isolate and followed by *S. epidermidis* [15]. But a recently study reported *Gram-positive bacteria* (64%) accounted for most episodes, followed by *Gram-negative bacteria* (26%) and *Candida* (10%) [12]. Another study reported the distribution of microorganisms' colonization on CVCs in Spain was as follows: *Gram-positive*, 68.4%; *yeasts*, 26.3%, *Gram-negative*, 5.3%; and with *S. epidermidis* predominately [10]. In addition, a study from Australia found that there were 136 diverse microbial genera detected on the IVC surfaces in children, and *Staphylococcus, Streptococcus,* and *Bacillus* predominate in the microorganisms [9]. Therefore, there are still some differences in epidemiology of microbial colonization presented in different studies. These differences may be attributed to different regions with different climates and different hospital environments which are directly related to bacterial colonization [16, 17]. Another possibility is that microbial flora has quietly changed and become diversity, no longer just *Staphylococcus*. Microbial diversity on the CVC surface should be focused, which needs us to pay more attention, strengthen monitoring, and even update epidemiological data.

The result of our study indicated that *Acinetobacter*, *S. epidermidis,* and *Candida albicans* were the most common colonies in CVCs. *Acinetobacter* spp. belongs to Gram-negative opportunistic pathogen and is an important nosocomial pathogen. *Acinetobacter* can be isolated from multiple parts of health human body [18] and reused medical devices. It could be found in many health care environment and causes human colonizer in hospitals, especially in patients with mechanical ventilation in intensive care units and indwelling catheters [19]. A retrospective study in an adult ICU in a tertiary care hospital has showed that most frequently isolated organism was *Acinetobacter baumannii* [20]. *Acinetobacter* spp. has become a global public health threat because of its increasing resistance to carbapenems and most other antimicrobial compounds [2]. *Acinetobacter* colonization in CVCs is the predominant microorganism in the study, which also may be related to the higher proportion of patients with CVCs from ICU in this study.

*S. epidermidis* is the second common isolated organism on CVCs in the study. *S. epidermidis* as symbiotic bacterium in human body is a parasite on the skin surface of human body [21]. *S. epidermidis* can be an opportunistic pathogen attaching to surfaces of medical implants and forms biofilm over indwelling catheters [22]. A small number of skin and mucosal bacteria contaminate the implanted catheters during surgical operation, which may also lead to bacterial colonization on the catheter surface. Therefore, CVC insertion provides a favorable entry and environment for the symbiotic bacterium on the skin surface. Therefore, health care providers should pay more attention and standardize insertion and maintenances of CVCs, which is a critical factor of CVC colonization.

In our study, *Candida* with 15.6% (59/379) was the third common organism and higher than that reported in the studies from Liu et al. [23] and Si et al. [24] and a similar recent study which reported *Candida* spp. with 15.5% [25]. *Candida* spp. has become the second and third frequent isolated species due to colonization on the CVC surface [10, 26], caused infection predominately among opportunistic fungal infections worldwide, and associated with high mortality rates [27]. Although there are differences in *yeasts* prevalence according to geographical differences, overall level worldwide is still growing [28, 29]. Furthermore, repeated exposure to broad-spectrum antibiotics, complex surgical procedures, long-term use of CVCs, hemodialysis catheters, corticosteroids, and toxic chemotherapeutic agents will increase the risk of fungal infections, especially *Candida*. From this, one knows that *Candida* colonization of CVCs with significant prevalence may continue to increase. However, whether they are the main microorganism causing catheter-related bloodstream infections needs further study.

We also found 107 (5.3%, 107/2020) CVCs were associated with a diagnosis of CLABSI. The most common organisms in causing CLABSI were *Acinetobacter* (23.4%), *S. aureus* (13.1%), and *Candida albicans* (12.1%). It is consistent with a study which reported the incidence rate of CLABSI is 4.3%–26% of placed catheters and 0.46–30 per 1000 catheter days but lower than results from Alonso et al. [10] and Khalil and Azqul et al. [30] presenting the CLABSI rate of 12.6% and 9.9%, respectively, and higher than a report with 3.9% rate of CRBSI from Cheng et al. who also reported *S. aureus* was the most common pathogens causing CLABIS [31]. However, a study carried out in China also found *Acinetobacter baumannii* (18.75%) was most common pathogen on intravascular catheters in ICU patients with catheter-related infection and followed by *S. epidermidis* [24]. Another study about pediatric patients found the most common pathogens of CLABIS were Enterobacteriaceae (36%), followed by *Gram-positive cocci* (29%), nonfermenting *Gram-negative bacteria* (16%), and *fungi* (16%) [25]. This variability is likely related to different variables as the characteristics of the patient population, the type of intravenous treatment (i.e., PN vs chemotherapy), and the nature of the environmental microflora [32]. In our study, *S. epidermidis* was the second common colonization bacteria on the CVC surfaces, causing CLABSI is less than *S. aureus*. It can be seen that the catheter colonization bacteria may be not necessarily related to CLABSI. *Acinetobacter* and *S. aureus* are common causative agent of infection on biomedical devices and harbor a variety of pathogenic tools with rapidly acquired resistance and mutation development which greatly increase mortality, morbidity, costs of treatment, and hospital stays. Therefore, catheter-related infections prevention and control not only requires attention to catheter microbial colonization, but also identification of catheter-related blood-borne infections, and their correlation may be different in disease progression.

There were also significant differences in the internal composition of isolated organisms on the catheter surface from different wards. The catheters from the pediatric ward with the highest rate of isolated organisms presented *S. epidermidis* predominately. This is consistent with Zhang's report [13], and their data show that the bacterial community of endovascular catheter in children was mainly *Staphylococcus*. Moreover, pediatric patients with imperfect immune system are more vulnerable to colonization of opportunistic pathogens from the skin surface. The positive rate of isolated organisms in the transplant ward was low, and no fungus was found, which may be related to high standard environment of ward and strict requirements of various medical techniques. Accordingly, iatrogenic factors may affect catheter microbial colonization and catheter-related infections to a large extent. Hospital managers need to strengthen environmental and personnel monitoring as to control iatrogenic factors and facilitate nosocomial infection control.

We also found isolated organisms with significant differences were existed in different years. The positive rate of catheter culture decreased, and the proportion of *Gram-negative bacteria* decreased gradually from 2013 to 2017. Besides, the trend of *Pseudomonas* proportion shows a gradual decrease among *Gram-negative bacteria*. However, similar studies analyzing *Pseudomonas* changing with years have not been reported. We guess that these trends may be related to the effectiveness of clinical prevention. As regards staphylococci, an increase in *S. aureus* colonization in 2015 was presented in the study but in 2016 with *S. epidermidis* predominantly. It may be due to CVCs from different wards (*X*^2^ *=* 3.939, *P*=0.047). Most of the positive-cultured CVCs with staphylococci were from general adult wards in 2015 and presented *S. aureus* predominantly. Conversely, in 2016, the positive-cultured CVCs with staphylococci were primarily from the intensive care unit, and neonatal unit presented *S. epidermidis* predominantly. A study from China has displayed *S. epidermidis* was the principal organism and responsible for neonatal sepsis [33]. *S. epidermidis* is one of the common biofilm-producing bacteria affiliating colonization on indwelling or implanted foreign bodies [34]. The proportion of other fungus except *Candida albicans* increased in 2017 (Figure 1). Although *Candida albicans* is the most common isolate of candidemia that has been recorded in study, this year's study also showed that these isolated fungus changed towards non-albicans *Candida* spp., such as *near-smooth Candida* and *smooth Candida* [29, 35]. With the widespread use of antibiotics, unfortunately, some of these species are naturally resistant to first-line antifungals which also make the prevalence of fungal infections to increase. More attention has been paid to *non-Candida albicans* fungal infections, and these fungal infections make treatment become more difficult [36]. It can be seen that the characteristics of microbial colonization of the central venous catheter change gradually, which needs health care providers pay more attention.

## 5. Conclusion

The study present and update epidemiological characteristics of microbial colonization on CVC surfaces and CLABSI. The prevalence of microbial colonization of CVCs is still significant which even has gradually changed over time, which will provide a reference for prevention and control of catheter-related infections in clinic.

## 6. Limitation

The study provides a new view about microbial colonization patterns in central venous catheters. However, it still has some limitations. One limitation was that all data about CVCs culture were derived from a general hospital only. Another limitation was that some information on certain drugs infusion, antibiotic sensitivities, and indwelling time of CVCs was not included into the analysis due to the retrospective nature of this analysis and imperfect electronic records. In future, it is necessary to improve the record of laboratory sample information and electronic records in clinic to promote future research in this field. Further studies based on multiple center, larger population, and more various factors of microbial colonization on CVC surfaces and CLABSI should be encouraged to guide clinical practice.

## Figures and Tables

**Table 1:** Basic characteristics of CVC sources (patients).

Items	Frequency, *N* = 2020	Percentages (%)
Age mean (range)	39.6 (0.01–94.00)	
Sex		
Male	1356	67.1
Female	664	32.9
Medical wards		
Pediatric ward	107	5.3
ICU ward	1177	58.3
Transplant ward	202	10.0
Other wards	534	26.4

CVC, central venous catheter.

**Table 2:** Etiology of the CVC colonization and CALBSI episodes.

Microorganism	Colonization (*n* = 379)	CALBSI (*n* = 107)
Gram-positive bacteria, *n* (%)		
*S. aureus*	25 (6.6)	14 (13.1)
*S. epidermidis*	43 (11.3)	7 (6.5)
*S. haemolyticus*	35 (9.2)	7 (6.5)
Other CoNS	31 (8.2)	7 (6.5)
*Enterococcus*	8 (2.1)	2 (1.9)
*Corynebacterium*	6 (1.6)	1 (0.9)
Others	4 (1.1)	1 (0.9)
Gram-negative bacteria, *n* (%)		
*Acinetobacter*	75 (19.8)	25 (23.4)
*Pseudomonas species*	37 (9.8)	12 (11.2)
*Klebsiella species*	14 (3.7)	3 (2.8)
*Colibacter*	12 (3.2)	3 (2.8)
*Enterobacter* spp.	11 (2.9)	5 (4.7)
Others	19 (5.0)	—
Fungi, *n* (%)		
*Candida albicans*	39 (10.3)	13 (12.1)
*Candida parapsilosis*	9 (2.4)	5 (4.7)
*Candida glabrata*	6 (1.6)	1 (0.9)
Others	5 (1.3)	1 (0.9)

CVC, central venous catheter; CLABSI, central line-associated bloodstream infection; CoNS, coagulase-negative staphylococci. The data in the table are presented as *n* (%), which refer to the number of isolated organism (*n*) and the percentage of different isolated organisms (%), respectively. The total number of isolated organisms was 379 (18.7%). *Gram-negative bacteria* with 44.4% were predominate among the total colonization bacteria on the CVC surfaces, followed by *Gram-positive bacteria* (40.1%) and *fungi* (15.6%). A total of 107 (5.3%) isolated organisms from CVCs were associated with a diagnosis of CLABSI. The most common organisms in causing CLABSI were *Acinetobacter* (23.4%), *S. aureus* (13.1%), and *Candida albicans* (12.1%).

**Table 3:** The prevalence of isolated organisms in different wards of catheters.

Species	Total	Wards (different sources of catheters)			
	(*N* = 2020) (%)	Pediatric ward (*N* = 107) (%)	ICU (*N* = 1177) (%)	Transplant ward (*N* = 202) (%)	Other wards (*N* = 534) (%)
Gram-positive bacteria	7.5	19.6	6.3	3.0	9.4
*S. aureus*	1.2	0.9	—	—	4.5
*S. epidermidis*	2.1	13.1	1.7	1.0	1.3
*S. haemolyticus*	1.7	1.9	2.1	—	1.5
Other CoNS	1.5	3.7	1.7	0.5	1.1
*Enterococcus*	0.4	—	0.6	—	0.2
*Corynebacterium*	0.3	—	0.2	—	0.6
Others	0.2	—	—	1.5	0.2
Gram-negative bacteria	8.3	1.9	5.9	2.5	17.2
*Acinetobacter*	3.7	1.9	2.3	1.0	8.2
*Pseudomonas species*	1.8	—	1.5	—	3.6
*Klebsiella species*	0.7	—	0.8	—	0.9
*Colibacter*	0.6	—	0.3	—	1.7
*Enterobacter* spp.	0.5	—	0.2	—	1.7
Others	0.9	—	0.8	1.5	1.1
Fungi	2.9	2.8	3.5	—	2.8
*Candida albicans*	1.9	1.9	2.4	—	1.7
*Candida parapsilosis*	0.4	—	0.5	—	0.6
*Candida glabrata*	0.3	—	0.4	—	0.2
Others	0.2	0.9	0.2	—	0.4

CVC, central venous catheter; PICC, peripherally inserted central catheter; ICU, intensive care unit; CoNS, coagulase-negative staphylococci. The data in the table presented refer to the prevalence of isolated organisms from CVCs. “*N*” refers to the total number of cultured catheters in different wards. If there were no isolated organisms after microbial culture, “—” is filled in the spaces. There were significant differences in isolated organisms on catheter surface from different wards (*X*^2^ = 124.046, *P* ≤ 0.001).

## Data Availability

The data used to support the findings of this study are available from the corresponding author upon request.
